# Science-based health innovation in Uganda: creative strategies for applying research to development

**DOI:** 10.1186/1472-698X-10-S1-S5

**Published:** 2010-12-13

**Authors:** Sheila Kamunyori, Sara Al-Bader, Nelson Sewankambo, Peter A Singer, Abdallah S Daar

**Affiliations:** 1 McLaughlin-Rotman Centre for Global Health, at the University Health Network and University of Toronto, MaRS Centre, South Tower, Suite 406, 101 College Street, Toronto, Ontario, M5G 1L7, Canada; 2 Makerere University College of Health Sciences, Kampala, Uganda

## Abstract

**Background:**

Uganda has a long history of health research, but still faces critical health problems. It has made a number of recent moves towards building science and technology capacity which could have an impact on local health, if innovation can be fostered and harnessed.

**Methods:**

Qualitative case study research methodology was used. Data were collected through reviews of academic literature and policy documents and through open-ended, face-to-face interviews with 30 people from across the science-based health innovation system, including government officials, researchers in research institutes and universities, entrepreneurs, international donors, and non-governmental organization representatives.

**Results:**

Uganda has a range of institutions influencing science-based health innovation, with varying degrees of success. However, the country still lacks a coherent mechanism for effectively coordinating STI policy among all the stakeholders. Classified as a least developed country, Uganda has opted for exemptions from the TRIPS intellectual property protection regime that include permitting parallel importation and providing for compulsory licenses for pharmaceuticals. Uganda is unique in Africa in taking part in the Millennium Science Initiative (MSI), an ambitious though early-stage $30m project, funded jointly by the World Bank and Government of Uganda, to build science capacity and encourage entrepreneurship through funding industry-research collaboration. Two universities – Makerere and Mbarara – stand out in terms of health research, though as yet technology development and commercialization is weak. Uganda has several incubators which are producing low-tech products, and is beginning to move into higher-tech ones like diagnostics. Its pharmaceutical industry has started to create partnerships which encourage innovation.

**Conclusions:**

Science-based health product innovation is in its early stages in Uganda, as are policies for guiding its development. Nevertheless, there is political will for the development of STI in Uganda, demonstrated through personal initiatives of the President and the government’s willingness to invest heavily for the long term in support of STI through the Millennium Science Initiative. Activities to support technology transfer and private-public collaboration have been put in motion; these need to be monitored, coordinated, and learned from. In the private sector, there are examples of incremental innovation to address neglected diseases driven by entrepreneurial individuals and South-South collaboration. Lessons can be learned from their experience that will help support Ugandan health innovation.

## Background

Uganda is a landlocked country in East Africa with a population of roughly 32 million [1]. After many years of political and economic instability, Yoweri Museveni became president in 1986 and began initiatives to reform the economy and rehabilitate the educational system, providing the foundation for the country’s current economic development. Predominantly agricultural, the economy has been growing steadily over the past five years. In 2009, the country’s GDP was $15.7 billion USD, and had been growing at over 6% annually for several years [2]. The service sector accounted for 52.8% of GDP while agriculture and industry accounted for 29% and 18.2% respectively [1].

Today the Ugandan government recognizes the need to use science and technology (S&T) as a means to achieve and maintain favourable socio-economic conditions for its population. It is investing in science education, and in 2006 began the $30m Millennium Science Initiative, a program to help improve human resources in S&T and to increase productivity in industrial, agricultural and other sectors using scientific tools and knowledge. Total government R&D spending nearly tripled from Uganda Shillings 31 billion ($19 million USD) in 2003/04 to approximately Uganda Shillings 82 billion ($47 million USD) in 2007/08, which accounts for about 0.4% of GDP in 2007/08 [3].

However, despite the improvement of some socio-economic indicators, Uganda is still ranked 156^th^ on the Human Development Index [4]. In 2008, life expectancy at birth was 53 years of age [2]. In common with other sub-Saharan African countries, health status remains poor. In 2002, HIV/AIDS, the largest disease killer, accounted for 25% of all deaths in the country followed by malaria and respiratory infections at 11% each, and tuberculosis at 4% [5]. Health expenditure by the government in 2007 stood at 2.1% of GDP and 27.9% of total public expenditure. However, with the current population size, the per capita public expenditure is only US$18. As a result, figures for 2007 show that 51% of health expenditure was private and out of pocket [6].

In this paper we present research on science-based health innovation in Uganda, including biotechnology. By science-based health innovation, we mean technological innovation across a spectrum of sophistication, from vaccines, pharmaceuticals, and medical devices to some plant medicines where attempts to scientifically standardize or characterize medicines have been made. We take a broad definition of innovation as not only new-to-the-world innovation, but also the diffusion, adaptation and use of technologies. We use the OECD definition of biotechnology: ‘the application of science and technology to living organisms, as well as the parts, products and models thereof, to alter living or non-living materials for the production of knowledge, goods and services’ [7].

The purpose of this paper is to describe and analyze science-based health innovation using an innovation system framework, which takes into account the wide variety of stakeholders who contribute to the innovation process and emphasizes the dynamic interaction and knowledge flow between them [8]. The study was undertaken at the invitation of the Vice President of Uganda, Professor Gilbert Bukenya. As far as the authors are aware, this is the first study of its kind on Uganda, with its emphasis on commercialization and understanding how knowledge translation happens in the area of health product development.

## Methods

A case study research methodology was used in this study [9]. Data was collected through reviews of academic literature and policy documents and through open-ended, face-to-face interviews in Uganda. Interviewees were identified through purposive and snowball sampling; we interviewed 30 people from across the science-based health innovation system, including government officials (n=8), researchers (n=13), private sector representatives (n=5), international donors (n=2) and non-governmental organization representatives (n=2) in February 2009. Since our initial research visits we have continued to engage stakeholders to address key challenges we identified, as we will discuss toward the end of the paper.

All quotes are from the interviews unless noted, and with permission. This study was approved by the Office of Research Ethics of the University of Toronto.

## Results and discussion

In the following sections, we describe and discuss Uganda’s science-based health innovation system.

### Government

At the government level, Science Technology and Innovation (STI) forms part of a number of different sector-based policies in Uganda. For example, the National Agricultural Research Policy of 2004 provides direction for agricultural research including biotechnology [10], and the National Industrialization Policy articulates the use of applied science research to develop Uganda’s infant industries [11]. In turn, these sector-based polices draw their priorities from national development policies such as the Poverty Eradication Action Plan (PEAP), the country’s strategic development plan guiding the formulation of government policy [12]. Each sector-based policy is managed by the relevant Ministry with apparently little coordination.

In the absence of a Ministry with responsibility for STI, the Uganda National Council for Science and Technology (UNCST), which sits within the Ministry for Finance, Planning, and Economic Development, is responsible for developing and advising the government on STI –particularly on integrating STI into the national development priorities, and for coordinating science research and development throughout the country.

UNCST’s priorities include improved agricultural productivity and human health. The Council has a Biotechnology Desk which, together with the National Biosafety Committee, is responsible for safe application of biotechnology. The National Biosafety Committee, which includes representatives from the scientific community, relevant ministries, farmers’ organizations and the private sector, drafted the National Biotechnology and Biosafety Bill which was approved by the Cabinet in April 2008. The objective of the Biotechnology/Biosafety Policy is to provide guidance on the development and application of biotechnology in Uganda; it aims to provide a regulatory and institutional framework for safe research and application of the technology. While this policy was formulated to support primarily agricultural biotechnology, these same institutions can also be used to support the development of health biotechnology. There is evidence that UNCST has made considerable efforts to coordinate efforts and policies among different institutions and has had some success; however, it remains limited by understaffing and its lack of status as a full Ministry.

The lack of a consolidated STI policy was considered by interviewees to be very problematic. From 1998, varying drafts of a national Science & Technology Bill have been brought forward to the Ugandan Cabinet but never fully adopted by government. In May 2009, Cabinet approved the latest draft of a Science, Technology and Innovation Bill, which was then to be debated in Parliament after revisions in the Attorney-General’s Office. If passed, it will be the first such S&T policy in Uganda that has gone through parliament [13]. Interviewees saw a need for such a policy framework, and expressed frustration at the lack of strong policy guidance to support their efforts.

All new drugs, vaccines, medical devices and diagnostics must be registered with the National Drug Authority (NDA), Uganda’s drug regulation agency, before they are licensed for use in the country. The NDA also performs Good Manufacturing Practice (GMP) audits of domestic and foreign manufacturers who seek to sell their products in the country. While the NDA was relatively well-regarded by interviewees, it faces institutional, human resources and funding challenges which are limiting its ability to work effectively. Funding from its responsible parent ministry, the Ministry of Health, was said to be irregular; the pressure to generate internal funds has led to charging manufacturers for regulatory checks, raising questions of potential conflicts of interest. Ability to regulate unfamiliar products is also a challenge: one interviewee cited the ‘huge problem in getting the NDA to recognize what GMP was in relation to diagnostics’. Another cited the lack of expertise to properly regulate health products as the “biggest frustration”. He suggested that “…the regulatory agency needs to be strengthened to allow them to do their regulating.” Guidelines to facilitate the task of regulating, which are based on WHO Best Practices and modified after discussions with local scientists and other stakeholders, remain in draft form awaiting gazetting, the last stage before they can become legal documents.

The framework to protect intellectual property in Uganda is through TRIPS (Trade-Related aspects of Intellectual Property Rights), which Uganda is subject to as a member of the World Trade Organization (WTO). Under TRIPS, Uganda is classified as a least developed country (LDC) and is therefore exempted until July 2016 from meeting certain obligations to implement the provisions. In moving towards TRIPS compliance, the Uganda Law Reform Commission was established to review laws and determine what was needed for compliance by 2016. As a result, bills like the Patents Amendment Bill (drafted 2000) and the Industrial Property Bill (drafted 2001), both of which make changes to the current patent legislation, have been drafted and are awaiting discussion in parliament. As an LDC, Uganda has opted for exemptions including permitting parallel importation and providing for compulsory licenses. Patent applications in Uganda are submitted through the African Intellectual Property Organization (ARIPO), an organization based in Zimbabwe established to pool the financial and human resources of its member countries in industrial property matters. Once approved by ARIPO, the patent is registered at the Registrar-General’s Office in the Ministry of Justice in Uganda; no examination takes place at the national level [14]. However, no locally-generated patents could be found in the health sector, and in general attitudes to patenting were ambivalent. One interviewee said ‘I don’t think in Uganda we are practicing it…I don’t know whether we have any institution for registering IP’.

Traditional medicine plays an important role in the healthcare system of Uganda, and more than 60% of the population depends on it due to low costs, easy access and cultural acceptance [15]. In 1999, a draft Traditional and Complementary Medicine Bill was presented to the Parliament; as of 2009, the Bill still had not passed and no policy developed for a strategic framework for traditional medicine. The Ministry of Health does however include traditional healers in its description of the private health sector in Uganda [16].

In the 2007/08 fiscal year, total R&D spending in the country (public and private sector) was ~0.4% of GDP and 1.82% of government expenditure [3]. This amount includes government spending of US$18 million, and direct donor contribution of US$22 million. However, as part of Uganda’s national budget is financed by donors, in reality the total donor contribution to R&D is higher. Of the total R&D amount, US$ 10.2 million was spent in Medical and Health Sciences R&D which amounts to 25% of total R&D spending.

Two interesting initiatives designed to bridge the gap between research and industrial application are the Millennium Science Initiative (MSI) and the Presidential Support to Scientists Fund. The MSI is a competitive scheme funded by the World Bank (US$30 million as a loan to the country on credit terms) and the Ugandan government (US$3.35 million). Administered by UNCST, MSI was started in the fiscal year 2006/07 for an initial four year period. MSI was originally envisaged as a biotechnology-specific funding initiative in four African countries (Cameroon, Bostwana, Namibia, Uganda). However, after extended discussions between the World Bank and the governments, the initiative was established only in Uganda and as a multi-science interdisciplinary initiative [17]. MSI has been divided into three categories or Windows. The first two, Windows A and B, are committed to institutional capacity building and research: Window A is grant funding for research teams and Window B provides funding to improve and/or create undergraduate programs in basic science and engineering. Grants in these categories have ranged from US$ 100,000 to US$ 800,000.

The third category in the MSI competitive grant, Private Sector Cooperation (or Window C), is a fund that aims to bridge the industry-research divide by providing monetary incentives for the two groups to collaborate. Two avenues exist for collaboration within this Window: Technology Platforms allow firms to identify a pressing technology problem and collaborate with researchers to address this problem, while Technology Internships provide funds for science and engineering students to formally intern at a firm in order to acquire hands-on experience in firm-level activities. Within the Technology Platforms, two stages of funding exist. The first stage, which has 10 two-year grants of US$50,000 each, enables firms and researchers to scout for possible technologies to address the problem. Once completed, the project team can apply for stage 2 funding (4 two-year grants of US$150,000) to pursue the research and adaptation needed. Between 2007 and early 2009, the Council had received about 45-50 proposals, of which four met the criteria and were funded for the stage 1 phase. A representative from UNCST highlighted that, despite being a competitive grant from across the private and public sectors, ‘our initial focus was to build national capacity, so there is a bias towards collaboration and team building rather than just an individual saying ‘I have an exciting innovation here’.

None of the four were in health or biotechnology; however the existence of the Window C fund indicates that opportunities exist for collaboration between firms and researchers in health and biotechnology. As a new and innovative initiative its progress should be monitored and evaluated to help learn lessons on how to support practical innovation activities.

The Presidential Support to Scientists Fund is the other government funding support for commercialization. Established in the fiscal year 2006/07 at the request of President Museveni, this fund provides scientists with support to take to market ideas that could be commercialized in the near term. Thus far, the Fund, with an annual budget of US$ 4.2 million [18], has supported five scientists to develop their innovations through proof of concept. All supported projects are food-related, such as fresh vacuum-packed green bananas which keep for one month (product samples have been sent to UK and Dubai). One of the projects, the production of a wine made from an indigenous plant “Murondo,” has also generated a patent. Although these are micro-scale projects, they indicate that entrepreneurialism can be stimulated, and that such initiatives may be candidates for scaling up. An understanding of the dynamics of innovation in these cases could help similar health-related initiatives.

Finally, to support the use of applied research and development in industry, the Ugandan government established the semi-autonomous government agency Uganda Industrial Research Institute (UIRI); UIRI was established in 1994 through an interest-free loan provided by the Chinese government. In 2003, a new director, Dr Charles Kwesiga, was appointed, and since then UIRI has grown. Of note is an incubator program which began in 2004 and provides support to small entrepreneurs. UIRI is currently incubating about eight projects in-house. Most of them are food-related and concerned with improving processes of manufacturing. Only one of them is health related (in animal health): a vaccine production laboratory which was scheduled to begin in mid-2009 production of a vaccine for highly contagious viral poultry disease called Newcastle disease, based on a heat-stable vaccine accessed through an Australian collaborator. UIRI networks with universities and with UNCST, though linkages are not yet strong – as one interviewee put it, ‘it’s a shaky bridge’.

### Research institutes and universities

Capacity to recruit and train science graduate students is essential to provide the human resources required for building up the health sector. Three of Uganda’s universities provide graduate training in science and technology: Makerere University, Mbarara University of Science and Technology, and Kyambogo University of Science and Technology, all of which are public universities. Graduate training in Health Sciences is concentrated at Makerere University’s College of Health Sciences (15 Masters programs and 15 PhD programs) and Mbarara University of Science and Technology Faculty of Medicine (9 Master’s programs and 2 PhD programs). Kyambogo has no Health Science programs but has 1 Master’s program in Natural Sciences [3]. Both Mbarara and Kyambogo are relatively new universities established, in 1989 and 2001 respectively, to meet the rising demand for scientists. Additionally, 90% of the government scholarships in public universities are geared towards scientists, with the remaining 10% reserved for students in the Arts [3]. Despite these efforts at increasing science capacity, interviewees indicated that lack of human capacity is a major impediment to innovation in the country.

Health research also takes place in public sector health research institutes. To coordinate all public sector health research in the country, the Uganda National Health Research Organization (UNHRO) was set up to act as Uganda’s equivalent of the US National Institutes of Health (NIH) – coordinating health research and receiving government and donor funds targeted at health research, thus promoting accountability for, and effective use of, funds. The government enacted UNHRO in May 2009, bringing the activities of the eight government health research institutes under one umbrella. (These eight institutes were the Uganda Virus Research Institute, Uganda Cancer Institute, Uganda Tuberculosis Investigation Centre, Natural Chemotherapeutics Lab, Central Public Health Laboratory, Uganda Trypanosomiasis Research Organization, Uganda Joint Clinical Research Centre, and Uganda Heart Institute). This is a promising development for Uganda.

#### Makerere University

Uganda’s largest and oldest university, Makerere University has approximately 30,000 undergraduate and 7,000 graduate students, and was the only public university until 1990 [14]. Established as a technical school in 1922, it achieved university college status first as part of the University of London in 1949, then as part of the University of East Africa in 1963. Makerere traditionally has specialized in medical and agricultural teaching and research [16]. A number of departments are involved in biotechnology research, including the College of Health Sciences and the Faculty of Veterinary Medicine within the Department of Parasitology and Microbiology. The College of Health Sciences has multiple international collaborations with institutes such as Johns Hopkins University (US), Yale University (US), Walter Reed Army Medical Centre (US) and Karolinska Institute (Sweden); these collaborations deepen its health research activities, particularly in HIV/AIDS and malaria.

In 2008 Makerere’s governing body approved an IP management policy to encourage its academics to commercialize their research, and a tech transfer office has been established in the university. So far there are no examples of health research commercialization for local application. One interviewee explained this by characterizing the type of research occurring as ‘operational research’, and maintained that donors, who fund the majority of health research, ‘are not interested in R&D in Uganda’.

An interesting initiative that has generated technology transfer, albeit of low-tech products, is the Innovations at Makerere (or I@Mak) program, funded by the Rockefeller Foundation. Some products that have been transferred to the community include a fuel-free incinerator that uses the gas byproduct of the burning waste to fuel the incinerator, and sanitary pads made from local plants that cost a fraction of the price of conventional pads. The incinerator has been taken up by the United Nations High Commission on Refugees, to be installed in refugee camps in the country, while the pads have been adopted by the United Nations Children’s Fund for distribution to schoolgirls to reduce interruptions to their daily attendance at school. Such initiatives illustrate a general decentralization process that seems to be occurring in Uganda, with universities becoming more linked into local civic and governance needs.

Of particular note is the Makerere University Private Sector Forum (MUPSF) which was established to create linkages between industry and the university. Specifically, the aim is to bring private sector leaders closer to the university in order to match industry need and university research and training. A major part of this initiative has been the establishment of honorary professorship positions at the University which have been awarded to five eminent private sector leaders. The initiative has been well received by the private sector leaders; one leader has proposed the establishment and funding of university chairs across various disciplines, and another leader has proposed establishing a research think-tank on economic policy. The Forum is currently focused on tailoring the university curriculum to meet the skills needs of the private sector, and it is just beginning to initiate dialogue among its members about industry-research linkages for commercialization.

#### Natural Chemotherapeutics Laboratories

Many interviewees identified traditional medicine as an area to focus on with respect to deepening Uganda’s research expertise. The Natural Chemotherapeutics Laboratory is an existing research facility under the Ministry of Health, which is responsible for providing science-based evidence to claims of efficacy of traditional medicine. The lab has a range of products in development, or in some cases being sold in the market. However no full-scale efficacy trials have been done and manufacturing capacity is limited.

To date, no firms have collaborated with the Laboratory. The low appreciation of commercialization is one reason why. One researcher commented that he recognized that he 'needs to overcome some inertia', which reflects hesitation to invest resources and time into the commercialization process – a process he sees as risky and unfamiliar. Another researcher commented that local researchers tend to 'go where things are moving faster' and prioritize projects that are well-funded, mostly international collaborations, while their own local research stagnates.

With the wealth of ongoing health research in the country, the two main challenges our study identified in Uganda’s research environment are the uncoordinated nature of the research efforts and the lack of human resources to carry out the research and development. Coordination has been difficult as the delay in enacting UNHRO created a vacuum in health research management and coordination. The lack of human resources is felt by all research institutes as the numbers graduating from the university programs are not enough to meet the demand for well-trained researchers.

Table 1 shows the products and processes being developed in Uganda’s research institutes.

**Table 1:** Products and Processes being developed in Uganda’s research institutes

Product	Health Area	Institute / Organization	Description
Aloe vera	Topical – for skin conditions	Makerere University	Traditional medicine. Available for use.
Aloe vera	Topical – for wounds	Natural Chemotherapeutics Laboratory	Traditional medicine – is said to promote synthesis of collagen. Available for use.
Avocine (avocado seed powder)	HIV/AIDS Malaria	Uganda Industrial Research Institute	Traditional medicine –A nutritional supplement. Sold on the market
Artemisia annua/elephant grass	Malaria	Natural Chemotherapeutics Laboratory	Traditional medicine - beverage preparation for treatment. In clinical trials
Peptide-based malaria vaccine	Malaria	Makerere University	Proof of concept successful but stagnated at next stage of development.
Rapid test for detection of multi-drug resistant tuberculosis (MDR-TB)	Tuberculosis	Makerere University	Enzyme-linked immunosorbent assay (ELISA) test for identifying MDR-TB in sputum; no incentive for commercialization

### Private sector

The presence of a pharmaceutical industry in a country is important for science-based health research and development to facilitate the final stage of the innovation cycle, especially for pharmaceutical products like drugs and vaccines. In Uganda, the pharmaceutical industry is small and focused on generics, manufacturing 10% of the drug products consumed in Uganda, with 90% imported mainly from India and China [19]. The Uganda National Drug Authority lists ten pharmaceutical firms in the country approved to manufacture generics. Some of these are building capacity in pharmaceutical manufacturing through South-South partnership agreements, and moving into areas of incremental innovation.

For example, Quality Chemicals, a domestic pharmaceutical firm, is the first firm to manufacture anti-retrovirals (ARVs) in Uganda. In 2005, Quality Chemicals established a partnership to produce ARVs with Indian generics manufacturer Cipla, where Quality Chemicals imports the active pharmaceutical ingredient from the Indian company. Cipla’s decision to partner with Quality Chemicals is partly due to the fact that Uganda’s IP regime takes advantage of a WTO waiver allowing poor countries to import generics of patented medicines without violating patent rights [20,21]. As of February 2009, the factory had scaled up to full commercial production, and since then has been manufacturing the triple-therapy generic ARV DUOVIR-N, which is a combination of three drugs in one tablet (Zidovudine, Lamivudine and Nevirapine). Quality Chemicals is also the first manufacturer in the country to achieve World Health Organization (WHO) GMP certification to enable them to be considered in international tenders.

The largest drug manufacturer in Uganda is Kampala Pharmaceutical Industries (KPI), owned by the Aga Khan Development Network, a collection of development groups active primarily in Asia and Africa. and with a mandate that includes supporting local industry development. KPI’s best known example of incremental innovation is Homapak, a chloroquine combination therapy developed as part of an integrated approach to address malaria in infants called “home-based management of fever”. Developed in partnership with the WHO and the Ministry of Health, homebased management involved both incremental technological innovation in the form of Homapack but also the social intervention of training drug distrubutors in local communities to treat children. This approach, was widely seen as an effective means to reducing infant mortality rates from malaria, and WHO recommended Homapak as the standard national treatment until 2006 when the world standard of malaria treatment changed to artemisinin. Again, KPI was the first in Uganda in 2006 to start making artemisinin combination therapies for malaria treatment through another technology transfer agreement with Cipla.

One of the main challenges found for firms was access to markets, with firms looking beyond the Ugandan market into the Democratic Republic of Congo (DRC), Southern Sudan, and Rwanda. However, infrastructure and fragmented delivery networks pose considerable problems both domestically and across countries, and the political instability of the two countries mentioned gave some interviewees cause for concern. Domestic firm-firm partnerships seemed to be very limited, with international linkages, particularly to India, more prevalent. ‘I think there is some suspicion’ between firms, was one interviewee’s interpretation.

Although technology transfer in Uganda is limited, there are some interesting activities worthy of note. One ‘virtual incubatee’ from UIRI is a health biotechnology enterprise called ASTEL. A local 8-10 person enterprise established by a Ugandan entrepreneur, Vinand Nantulya, ASTEL has just begun manufacturing diagnostic kits for malaria, hepatitis B and pregnancy using lateral flow technology. This technology is not novel globally but it is new to the country, and extremely beneficial in situations where populations live far from test sites as lateral flow diagnostic tests significantly reduce waiting time for results. ASTEL has no local competitors in Uganda, and is priced competitively compared to imports. Dr Nantulya intends to start substituting raw materials for locally-made ones, and thus, in time, to drive price down further.

While significant novel R&D is not taking place within firms in Uganda, it is the goal of a private biomedical research institution, Med Biotech Labs, which was started in 1995 by Dr Tom Egwang, a Ugandan microbiologist. The mission of Med Biotech is to undertake research on diseases that affect the Ugandan population, to build capacity in biotechnology and medical research and to facilitate technology transfer. Med Biotech engages in R&D, and preclinical and clinical trials of medical and agricultural biotechnology. Under an MSI competitive grant, Med Biotech won US$800,000 to prepare infrastructure for malaria vaccine clinical trials set to take place in Northern Uganda, and to fund capacity building for these trials. This grant is the first major funding Med Biotech has received from the Uganda government. Other sources of funding for Med Biotech include an NIH planning grant to build capacity for malaria research, and European Union funds for collaboration with European institutes in malaria vaccine discovery. This extensive funding support has enabled Med Biotech to undertake innovative dual-purpose projects, such as the malaria vaccine clinical trials in Northern Uganda which our interviewee said are also intended to draw resources to a very underdeveloped part of the country.

The absence of early-stage finance and human resources were reported as hindering innovation and longer-term strategies. We found no evidence of venture capital for health innovation during our study and identified only one instance of angel funding from private sources. No interviewee asked could provide examples of venture capital pools or other organized private sources of early stage funding. Government support for small enterprises is minimal in this respect. However, it is encouraging that MSI money is beginning to be disbursed towards more innovative health enterprises and the element of competition among firms or NGOs may be productive in generating novel ideas and flexible business models. As in the public research institutes, inadequate technical skills were also mentioned as a constraint to private sector R&D. Though KPI has a lab outfitted to conduct R&D, our interviews indicate that insufficient human resources mean that the lab is used for drug formulation only, and no R&D takes place within the firm. To meet its human resource needs, Med Biotech Labs has had to train its own personnel, and does so by taking in PhD and Masters students and giving them on-the-job training. This supports one interviewee’s assertion that local human resources ‘can be harnessed’ if enterprises are given support.

### NGOs and donors

As indicated in earlier sections, foreign donors – such as the Rockefeller Foundation, the World Bank and the European Union - have been instrumental in almost all of the innovation activities in Uganda, and as such strongly influence the health innovation landscape. We also identified two other donor-driven initiatives aimed specifically at strengthening commercialization.

The first one, BIO-EARN, is a regional project funded by the Swedish International Development Co-operation Agency (SIDA) that aims at increasing research capacity primarily in agricultural, environmental and industrial biotechnology. The program is a regional one and includes researchers from Kenya, Uganda, Tanzania and Ethiopia. The BIO-EARN project also has a commercialization component whose objective is to encourage linkages between industry and research: in 2008 grants were competitively awarded to six projects which relate to environmental, industrial and agricultural biotechnology to encourage developing their technologies into products with the help of industry [22]. A recent evaluation of the project drew a number of lessons, including the need to take into account both economic and social demand for biosciences knowledge; the need for product development funding to be included within grants, regardless of whether the knowledge will be disseminated in a market or non-market context; and the need to bring together wider expertise than only science within biosciences research projects [22]. SIDA also funds health research capacity building, and as of 2008 was funding 35 PhD students in the Makerere University College of Health Sciences on projects ranging from reproductive health to molecular biology research [19].

The United States Agency for International Development (USAID), through the Program for Biosafety Systems, supports the development of biotechnology and biosafety policies. They have also funded the activities of the Uganda Legal Reform Commission, established to evaluate the changes needed to Uganda’s legal framework to become TRIPS-compliant.

An interesting initiative by a domestic institution is also playing a part in Uganda’s innovation system. In September 2008, the Uganda National Academy of Sciences (UNAS) started a pilot pairing scheme between MPs who sit on the parliamentary science and technology committee and Ugandan scientists. UNAS is a non-governmental entity that provides a forum for the advancement of science through discussion and debate. Modeled on a similar UK pairing project, five Ugandan scientists were matched with five Ugandan legislators. The scientists spent a week in Parliament shadowing the partner parliamentarian. Conversely, the parliamentarians were to visit the scientists at their respective research institutes. The project proved to be successful in its objective of sensitizing each partner in the pairing, and the project was seeking funding to scale up in 2009.

## Conclusions

### Strengths and good practices

Our case study highlighted several strengths which Uganda could build upon to improve its health product innovation system. Among them are: political will to developing STI as shown by the government’s investment in the MSI program and the Presidential Support to Scientists Fund; the presence of health research in-country particularly at Makerere University College of Health Sciences, Med Biotech Labs and at the public health research institutes; a pharmaceutical industry that has started to create partnerships, particularly South-South ones which encourage health product innovation and are beginning to think of access to markets beyond Uganda; and a traditional medicine research base particularly at the National Chemotherapeutics Labs which has begun to explore the commercialization of Uganda’s indigenous knowledge. A variety of support mechanisms are also coming into existence to encourage innovation, such as small-scale incubators, and public-private fora. How these will be coordinated in the future is an important question.

Unique to Uganda is the MSI Private Sector Cooperation Fund (Window C Fund). Through our interviews we learned that initial response to this initiative has been slow: between 2007 and early 2009, the Council received 45-50 proposals of which only four met the criteria and quality required. UNCST staff attributed the low response and poor quality of the proposals to a number of reasons. Firstly, both academics and the private sector are overly cautious, sometimes even suspicious, when interacting with each other. Secondly, the private sector tends to have a shorter term perspective on the value of R&D to its profits than is needed for health product development; it has therefore been reluctant to invest time and money to cover what costs may not be eligible under the grant into this collaboration. Thirdly, it seemed that the private sector firms that did get involved were not clear about their role within the collaboration, which has led to weaker ownership from the private sector partner. All the proposals that are being funded were prepared and led by the academic partner in the collaboration, and therefore from UNCST’s perspective Window C has not quite achieved the intended degree of collaboration. In response to these early lessons learned, UNCST and UIRI are working jointly to increase awareness in industry and engage the private sector. UIRI is helping to disseminate information and run workshops which include information on how to develop good proposals. The two agencies have also produced the MSI Window C Manual to encourage and educate the private sector on its role and the payoff in supporting R&D.

Despite its slow start, we consider the Window C Fund an important, promising component of the innovation process in Uganda. In addition to providing the financial means for a concept to be tested, this Fund encourages collaboration between firms and researchers through the exploration of a real problem experienced by industry. Such opportunities for collaboration in turn increase awareness in researchers about commercialization and appreciation in the private sector for R&D. Most importantly, as this fund is one of the few product development funds currently in operation in sub-Saharan Africa, the lessons learned in this case could be built upon by other African countries seeking to implement this important component of the innovation process.

### Recommendations

Science-based health innovation is nascent in Uganda. The political will to develop STI capacity, demonstrated through initiatives such as the Presidential Support to Scientists Fund, has made some progress in stimulating commercialization. However, government leadership at the policy level is needed to align the priorities and expertise of research institutions and universities with the nation’s priorities. This gap is evident in two areas. Firstly, in the absence of a ministry responsible solely for STI, key players in the Ugandan government are scattered through a number of ministries. UNCST is therefore limited in delivering on its mandate to coordinate. From our study it is evident that this fragmentation reduces the ability of the different players to coordinate science research towards commonly understood national priorities, and sometimes the different agencies created have parallel mandates which creates friction amongst them. Secondly, even with strong political support for STI, a major stumbling block is the stagnation of Bills in the parliament or the cabinet as they await debate and approval. Examples of policies that are stuck include the STI policy (presented to Cabinet in 2006 and as of May 2009, has been passed in Cabinet but not in Parliament), the Traditional Medicines Bill (presented to Cabinet in 2000 but not yet passed),and the Bill to enact UNHRO (presented to Cabinet in 2001 and finally approved by Cabinet and Parliament in 2009). While the existence of these draft policies indicates definite awareness of the policies needed to support STI, particularly health innovation, the stagnation of the bills has slowed down the process of strengthening the policy framework. Due to this gap in government leadership, local health research priorities are more often than not responsive to foreign organizations and not directed towards local industry needs and demand.

A range of activities to support innovation have been put in motion, such as the creation of incubator facilities and tech transfer initiatives like the I@Mak program, which demonstrates not only attention to the impact of knowledge but a genuinely new institutional approach to meeting economic and social demands at a district level. In the private sector, there are examples of incremental innovation to address neglected disease, driven by entrepreneurial individuals. What is needed is linking the different innovation initiatives more effectively.

Insufficient human resources, particularly in research institutes and pharmaceutical firms, are an impediment to health innovation. It was perceived by our interviewees that not enough high quality scientists are graduating from the local university system, which in turn means that research institutes do not have sufficient research expertise. For example, only 40 pharmacists graduate every year from the training programs. Firms struggle with finding the industrial skills base they need. Within government, lack of expertise in R&D regulation has also slowed down some innovation initiatives.

Our study found some linkages exist between research, the private sector and government to enable development of novel health solutions and products, and that limited interactions between the different groups do take place. Among other examples, UIRI networks with universities and the MUPSF supports private-public collaboration. However, these linkages are not yet leading to increased and efficient knowledge flows between industry and research that support commercialization.

*Recommendation 1: Create a Ministry of Science, Technology and Innovation.* The creation of a Ministry of Science, Technology and Innovation was suggested by interviewees in our study, as well as in a number of research studies on Uganda and in commentaries in local newspapers. Many see this Ministry as a critical missing piece for improving innovation in Uganda, and the latest version of the STI policy does includes the creation of this Ministry as a part of the implementation. The Ugandan government also needs to expedite the parliamentary process of approving policies to enable the implementation of this recommendation. To alleviate this delay, initiatives such as the parliamentarian-scientist pairing piloted by the Uganda National Academy of Sciences in 2008 can be scaled up to include and sensitize more parliamentarians. In the UK, a similar initiative has resulted in a greater understanding by parliamentarians of the need for the specific policies that support STI. A similar outcome is possible in Uganda.

*Recommendation 2: Support human resource development in health innovation* by capitalizing on and supporting existing skills training and transfer programs. Some initiatives to increase training are already in place, and indicate how meeting the human resource gap in research institutes and in firms could be achieved. MSI’s Window C provides an opportunity for a demand-driven training process where training is done to meet industry demand. In the private sector firms, programs exist to train university students in pharmaceuticals through internships and to provide hands-on industrial training. These demand-driven training programs ensures that university graduates are trained in skills currently needed in industry, thus reducing the skills gap more effectively. Such initiatives should be sustained and scaled up, in addition to paying attention to the more traditional training at universities.

*Recommendation 3: Monitor, evaluate, and disseminate lessons from innovative initiatives* by creating means to identify and understand their successes and challenges. These initiatives need to be monitored and coordinated in order to learn lessons that are specific to the Ugandan context. Opportunities to learn lessons on how to support Ugandan health innovation and to build on their pioneering efforts are currently being missed. Such monitoring and evaluation processes would also ensure that innovative efforts are not being duplicated.

*Recommendation 4: Create a mechanism to enhance knowledge translation and linkages between business, science and capital providers in the health area.* Platforms such as MUPSF indicate nascent linkage mechanisms exist in Uganda, but they need to work within a larger context of knowledge flows between research and industry with the specific aim of commercialization and a focus on clear health targets. Uganda could capitalize on these existing methods as a starting point for building partnerships that could lead to collaboration. In addition, as noted in our previous work, life sciences innovation centers are another mechanism for focusing linkages around innovation and product development [23-25]. Developing this mechanism could facilitate funding flows, particularly from foreign sources. Scaling up of funding support for commercialization like the MSI program and the Presidential Support to Scientists Fund would encourage more technologies to move out of the labs and into the marketplace. Given the restricted domestic capital options in Uganda, stronger linkages with foreign sources could potentially bring in “patient capital” that might offer funds for R&D in exchange for a reasonable financial return.

Uganda, because of its history of high quality health research at Makerere University and other research institutes, has a sound basis for going to the next level: to capture more of the value of its research and, working with the private sector and other partners, to focus on health product development and translation of research into applied solutions.

Science-based health product innovation is in its early stages in Uganda, as are policies for guiding its development. Nevertheless, Uganda has recently been at the forefront of interesting new approaches to S&T development, particularly with respect to generating innovation and local value from research in a variety of ways. Experience from other countries and contexts has demonstrated how technological innovation requires institutional innovation – Uganda’s activities to support technology transfer and private-public collaboration need to be monitored, coordinated, and learned from over coming years, so that it and other countries in Africa can make health and biomedical knowledge work for them.

## Competing interests

None declared.

## Authors' contributions

SK, SAB, NS, ASD and PAS contributed to the concept and design of this study, participated in site visits, collected and analyzed the data, and participated in manuscript development.
